# The predictive value of MAPH score for determining thrombus burden in patients with non-ST segment elevation myocardial infarction

**DOI:** 10.1186/s43044-022-00299-1

**Published:** 2022-08-15

**Authors:** Özge Çakmak Karaaslan, Cem Çöteli, Murat Oğuz Özilhan, Ahmet Akdi, Funda Başyiğit, Hatice Selçuk, Mehmet Timur Selçuk, Orhan Maden

**Affiliations:** 1 Department of Cardiology, Ankara Bilkent City Hospital, Health Sciences University, Ankara, Turkey; 2 Department of Cardiology, Faculty of Medicine, Ankara Yıldırım Beyazıt University, Ankara, Turkey

**Keywords:** Blood viscosity, MAPH score, NSTEMI, Thrombus burden

## Abstract

**Background:**

A high thrombus burden has been connected with poor clinical events in patients with non-ST segment elevation myocardial infarction (NSTEMI). In patients with STEMI, a high MAPH score has been associated with a large thrombus burden. However, the predictive value of the MAPH score in determining the thrombus burden in patients with NSTEMI is unclear. The present report aimed to evaluate the prognostic role of the MAPH score in the estimating coronary thrombus burden in NSTEMI patients. The study patients were split into two groups according to their thrombus grade. The low shear rate (LSR) and high shear rate (HSR) were estimated by haematocrit levels and serum total protein levels. The MAPH score was calculated by adding mean platelet volume (MPV) levels and age, in addition to total protein and haematocrit.

**Results:**

The patients with a high thrombus burden (HTB) had a higher LSR, higher HSR and higher MAPH score compared to patients with low thrombus burden. MAPH score was found to be an independent predictors of HTB in Model 1 (OR: 1.124, 95% CI: 1.011–1.536; *p* = 0.039) and Model 2 (OR: 1.236; 95% CI: 1.002–1.525; *p* = 0.047). The cut-off value of the MAPH score for predicting HTB was 2 based on the Youden index.

**Conclusions:**

The MAPH score, which calculated by adding MPV levels and age, in addition to total protein and haematocrit, is a novel, easily accessible score. The MAPH score at both LSR and HSR was an independent predictor of HTB.

## Background

Non-ST segment elevation myocardial infarction (NSTEMI) is the most widespread subcategory of acute coronary syndromes and is a major reason of morbidity and mortality [1]. As the incidence of NSTEMI has increased in the last two decades, risk stratification of these patients is important in predicting poor clinical outcomes [2]. A high thrombus burden (HTB) is related to adverse complications such as distal embolization, no reflow and stent thrombosis in patients with NSTEMI [3].

The MAPH score is a straightforward tool used to forecast thrombus load in patients with ST-segment elevation myocardial infarction (STEMI) [4]: a high MAPH score is strongly related to an HTB in patients with STEMI [4]. However, the predictive value of the MAPH score of determining thrombus burden in patients with NSTEMI is unclear. The present study purposed to assess the prognostic function of the MAPH score in the prediction of coronary thrombus burden in patients with NSTEMI.

## Methods

### Study design and study population

The study population contained 743 consecutive patients admitted with NSTEMI at our hospital between May 2020 and April 2021. The institution registers and patient files were revised. We excluded 121 patients according to the following criteria: known atrial fibrillation; chronic inflammatory disease, neoplasms or severe hepatic disease; end-stage renal disorder (estimated glomerular filtration rate < 30 ml/min/1.73 m^2^); undergoing renal replacement therapy; no intracoronary lesions shown via angiography; history of cardiac valve surgery; using oral anticoagulants; patients with underwent coronary angiography (CAG) after 24 h of admission with a diagnosis of NSTEMI, and missing clinical data. This study was confirmed by our medical institution ethics committee in compliance with the Declaration of Helsinki.

The remaining 622 NSTEMI patients who performed coronary angiography were enrolled in the retrospective study.

### Definitions

NSTEMI was described as the existence of clinical symptoms in accordance with myocardial ischaemia detected by anomalous cardiac enzymes without persistent ST segment elevation [5].

Diabetes mellitus (DM) was identified as fasting glucose levels over 126 mg/dL or glucose levels more than 200 mg/dL at any time measurement or, usage of antidiabetic drugs. Hypertension was identified as resting blood pressure higher than 140/90 mm Hg or current usage of antihypertensive drugs. Hyperlipidemia was identified as total cholesterol higher than 200 mg/dL and/or low-density lipoprotein cholesterol more than 140 mg/dL. Stroke was identified as a known ischaemic stroke or transient ischaemic attack. Congestive heart failure (CHF) was identified as a known heart failure symptoms affirmed with reduced left ventricular ejection fraction (LVEF).

### Laboratory measurements

White blood cell counts were studied with an automated haematology analyser XE-1200 within 30 min of blood sampling, and biochemical measure was analysed with a molecular analyser at the hospital’s biochemistry laboratory. The biochemical variables were calculated by conventional methods.

For all patients, the LSR and HSR were measured by the haematocrit and total protein utilizing the previously validated formulation of Simone et al. [6]. The cut-off levels for MPV, age, total protein and haematocrit were calculated according to the Youden index for HTB. Levels higher than the cut-off were accepted as a score of 1, and MAPH score was evaluated accordingly [4].

### Angiography analysis

Coronary angiography (CAG) was carried out utilizing the Judkins technique through the transfemoral or transradial approach. The precise timing of the CAG was at the discretion of the primary physician’s judgement. In accordance with the current guidelines [2], an immediate invasive intervention was made on patients with NSTEMI with at a minimum one very high risk criterion; all the remaining patients performed coronary angiography within 24 h of admitting with a diagnosis of NSTEMI.

The study population who performed percutaneous coronary intervention (PCI) received 70–100 U/kg intravenous unfractionated heparin before the PCI. The PCI procedures were performed using the transfemoral or transradial approach. The study population received a loading dose of aspirin and, depending on the operator’s decision, a loading dose of ticagrelor 180 mg, clopidogrel 600 mg or prasugrel 60 mg after the selection to proceed with PCI was made. Procedural decisions about adjunctive pharmacotherapy, such as glycoprotein IIb/IIIa inhibitors, were made by the operator.

Thrombus grades were categorized into five stages as according to the reports of Gibson et al. [7]. A case was considered to have angiographically obvious thrombus if TIMI thrombus grades 2–5 were an existing. TIMI thrombus grade 0, no cine angiographic images features of thrombus were available; TIMI thrombus grade 1, possible thrombus was available, with such angiography features as decreased contrast intensity, hazy images, disordered contour; TIMI thrombus grade 2, there was precise thrombus, including the biggest dimensions ≤ 1/2 the coronary artery diameter; TIMI thrombus grade 3, there was precise thrombus but including maximal linear dimension > 1/2 but < 2 the coronary artery diameters; TIMI thrombus grade 4, there was precise thrombus, including the greatest dimension ≥ 2 coronary artery diameters; TIMI thrombus grade 5, there was total occlusion. The TIMI thrombus grades were calculated separately for each case from the diagnostic CAG obtained before to PCI. Low thrombus burden (LTB) was described as TIMI thrombus grade 0–3, and HTB was described as TIMI thrombus grade ≥ 4. The analysis patients were separated into LTB and HTB groups based on thrombus burden.

Two cardiologists who were blinded to the MAPH score of study population analysed the study patients’ angiographic data. In case of inconsistency between the two cardiologists, a third reviewer’s idea was taken.

### Statistical analysis

All parameters were studied usage the SPSS 22.0 Statistical Package Program for Windows (SPSS; IBM, Armonk, New York, USA). The categorical data between groups were compared usage the *χ*^2^ test or Fisher’s exact test. The Kolmogorov–Smirnov test was employed to evaluate the normality of distribution. The continuous variables were displayed as mean ± standard deviation or median ± interquartile ranges, and the categorical variables were displayed as the number of patients and percentages. A comparison between study populations was performed with the Student’s t test for the normally distribution and the Mann–Whitney U test for the skewed distributed variables. A value of *p* < 0.05 (using a two-sided test) was adopted as statistically significant. Multivariate logistic regression analyses were performed to assess the predictors of HTB. Two unlike models were designed to evaluate the predictiveness of MAPH score at each shear rate of whole blood viscosity (WBV) levels at multivariate analysis. ROC analyses were made to evaluate the predictive value of variables for HTB. The cut-off value was considered based on Youden index.

## Results

The final study population consisted of 622 NSTEMI patients. The study population was separated into LTB and HTB groups based on thrombus burden: LTB (*n* = 339) and HTB (*n* = 283). The baseline characteristics and laboratory parameters of the study groups according to TIMI thrombus grade are shown in Table 1. The mean age was 62 ± 18 years, and most study population (74.3%) was male gender. The patients with HTB had higher ages (*p* = 0.035), lower LVEF (*p* = 0.009), a higher prevalence of DM (*p* = 0.010), higher prevalence of CHF (*p* = 0.038) than the patients with an LTB.

**Table 1:** Baseline characteristics and laboratory values of the study groups according to TIMI thrombus grade

	All Group (*n* = 662)	LTB (*n* = 339)	HTB (*n* = 283)	*p* value
Age (year)	62 ± 18	61 ± 18	63 ± 17	0.035*
Male, *n* (%)	462 (74.3)	245 (72.2)	217 (76.6)	0.231
LVEF, %	50 ± 8.5	50 ± 7	50 ± 5	0.009*
Diabetes mellitus, *n* (%)	286 (46)	140 (41)	146 (52)	0.010*
Hypertension, *n* (%)	374 (60)	196 (58)	178 (63)	0.218
Hyperlipidemia, *n* (%)	260 (42)	134 (40)	126 (45)	0.208
Previous stroke, *n* (%)	38 (6)	22 (7)	16 (6)	0.665
Congestive heart failure	114 (18)	52 (15)	62 (22)	0.038*
Previous history of CAD	214 (34)	125 (37)	89 (31)	0.175
Glucose, mg/dl	119 ± 82	111 ± 62	129 ± 95	< 0.001*
Creatinine, mg/dl	0.9 ± 0.2	0.8 ± 0.2	0.9 ± 0.2	0.035*
LDL-C, mg/dl	110 ± 52	107 ± 50	112 ± 51	0.048*
HDL-C, mg/dl	35 ± 10	36 ± 11	35 ± 10	0.069
Triglyceride, mg/dl	130 ± 83	138 ± 102	123 ± 71	0.148
Total cholesterol, mg/dl	174 ± 52	173 ± 52	178 ± 51	0.301
Total protein, g/l	66 ± 8	65 ± 6	67 ± 9	0.001*
Albumin, g/l	41 ± 4	42 ± 4	42 ± 5	0.396
Haemoglobin, mg/dl	14.1 ± 2.3	13.8 ± 2.6	14.3 ± 2.4	0.001*
Haematocrit, %	41.8 ± 6.5	40.9 ± 7.0	42.6 ± 6.9	< 0.001*
Neutrophil, 10^3^ μl	6.3 ± 3.7	5.9 ± 3.2	6.7 ± 4.1	< 0.001*
Lymphocyte, 10^3^ μl	1.8 ± 0.8	1.8 ± 0.9	1.8 ± 0.8	0.746
Platelet, 10^3^ μl	258 ± 95	256 ± 95	259 ± 96	0.676
WBC, 10^3^/µl	9.3 ± 4.1	9.0 ± 3.7	9.8 ± 4.1	< 0.001*
MPV	8.2 ± 1.3	8.1 ± 1.0	8.3 ± 1.5	0.001*
CRP (mg/L)	14 ± 9	13 ± 8	15 ± 10	0.018*
Troponin (ng/L)	7657 ± 725	7269 ± 5664	8105 ± 9964	0.001*
LSR	33.8 ± 31.6	27.0 ± 26.1	42.4 ± 34.0	< 0.001*
HSR	15.9 ± 1.2	15.7 ± 1.1	16.3 ± 1.3	< 0.001*
MAPH	2.2 ± 0.9	2.0 ± 0.9	2.4 ± 0.9	< 0.001*

Data are presented as mean ± standard deviation for normal distribution or median ± interquartile range for not-distribution normality or *n* (%)

*LTB* low thrombus burden; *HTB* high thrombus burden; *LVEF* left ventricular ejection fraction; *CAD* coronary artery disease; *LDL-C* low-density lipoprotein cholesterol; *HDL-C* high-density lipoprotein cholesterol; *WBC* white blood cell; *CRP* C-reactive protein; *LSR* low shear rate; *HSR* high shear rate

*A value of* p* < 0.05 is statistically significant

HTB patients had higher glucose (*p* < 0.001), creatinine (*p* = 0.035), low-density lipoprotein cholesterol levels (*p* = 0.048), total protein (*p* = 0.001), haemoglobin levels (*p* = 0.001), haematocrit (*p* < 0.001), neutrophil (*p* < 0.001), MPV (*p* < 0.001), C-reactive protein (*p* = 0.018) and troponin levels (*p* = 0.001) and higher white blood cell counts (*p* < 0.001) compared to patients with an LTB.

As expected, the patients with an HTB had a higher LSR (*p* < 0.001), HSR (*p* < 0.001) and MAPH score (*p* < 0.001) compared to patients with LTB (Table 1).

The study population’s angiographic data are shown in Table 2. There were no significant differences between the study groups in terms of culprit vessels. In terms of revascularization, the ratios of patients who underwent coronary artery bypass grafting or PCI were similar in both groups. The number of patients undergoing medical therapy after CAG was higher in the LTB group (*p* < 0.001) (Table 2).

**Table 2:** Angiographic data and selection of revascularization of study groups according to TIMI thrombus grade

	All group (*n* = 622)	LTB (*n* = 339)	HTB (*n* = 283)	*p* value
*Angiographic data, n (%)*				
Culprit vessel, *n* (%)				
Left main	15 (2.4)	9 (2.7)	6 (2.1)	0.795
Left anterior descending	254 (40.8)	144 (42.5)	110 (38.9)	0.369
Left circumflex	167 (26.8)	87 (25.7)	80 (28.3)	0.469
Right coronary artery	139 (22.3)	75 (22.1)	64 (22.6)	0.923
Bypass graft	47 (7.6)	24 (7.1)	23 (8.1)	0.650
*TIMI thrombus grade scale, n (% in all patients)*				
0	122 (19.6)	122 (36)		
1	40 (6.4)	40 (11.8)		
2	71 (11.4)	71 (20.8)		
3	106 (17)	106 (31.3)		
4	177 (28.5)		177 (62.5)	
5	106 (17)		106 (37.5)	
*Selection of revascularization, n (%)*				
Coronary artery bypass grafting	39 (6.3)	20 (5.9)	19 (6.7)	0.741
Percutaneous coronary intervention	563 (90.5)	305 (90)	258 (91.2)	0.681
Medical therapy after coronary angiography	13 (2.1)	13 (3.8)	0	< 0.001*

Data are expressed as number (percentage)

*TIMI* thrombolysis in myocardial infarction

*A value of* p* < 0.05 is statistically significant

We established two different analysis models to evaluate the predictability of MAPH score at separately shear rate of WBV level, using multivariate analysis (Table 3). In multivariate analysis, the MAPH score (Model 1, odds ratio [OR] = 1.124, 95% confidence interval [CI] = 1.011–1.536; *p* = 0.039), LSR (Model 1, OR = 1.011, 95% CI = 1.004–1.019; *p* = 0.003), and neutrophil count (Model 1, OR = 1.564, 95% CI = 1.021–3.396; *p* = 0.040) were found to be independent predictors of an HTB. Model 2 shown that MAPH score (Model 2, OR = 1.236; 95% CI = 1.002–1.525; *p* = 0.047), HSR (Model 2, OR = 1.233, 95% CI = 1.091–1.485; *p* = 0.002), and neutrophil count (Model 2, OR = 1.568, 95% CI = 1.021–2.408; *p* = 0.040) were as independent predictors of an HTB (Table 3).

**Table 3:** Multivariate logistic regression analysis for prediction of high thrombus burden

	Odds ratio (95% CI)	*p* value
*Model 1*		
MAPH score	1.124 (1.011–1.536)	0.039
LSR	1.011 (1.004–1.019)	0.003
WBC	0.722 (0.479–1.089)	0.120
Neutrophil	1.564 (1.021–2.396)	0.040
Lymphocyte	1.399 (0.828–2.363)	0.209
*Model 2*		
MAPH score	1.236 (1.002–1.525)	0.047
HSR	1.233 (1.091–1.485)	0.002
WBC	0.720 (0.476–1.087)	0.118
Neutrophil	1.568 (1.021–2.408)	0.040
Lymphocyte	1.398 (0.822–2.359)	0.218

*CI* confidence interval; *LSR* low shear rate; *WBC* white blood cell; *HSR* high shear rate

**Fig. 1:**
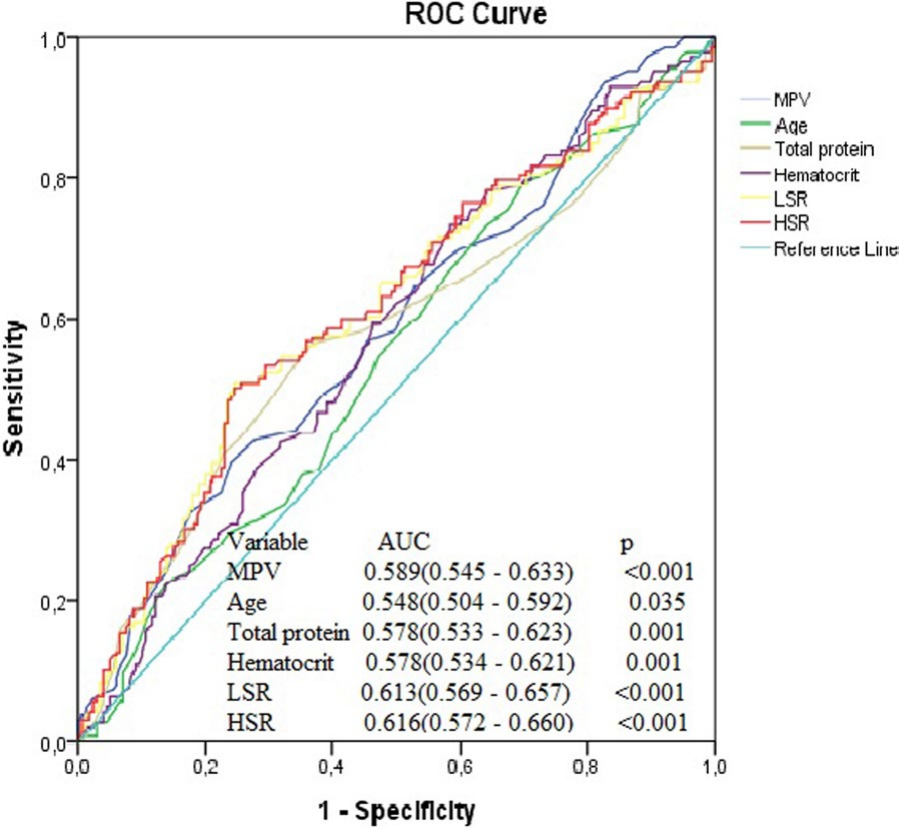
Receiver operating characteristics curve analysis of MPV, age, total protein, haematocrit LSR and HSR in predicting high thrombus burden

**Fig. 2:**
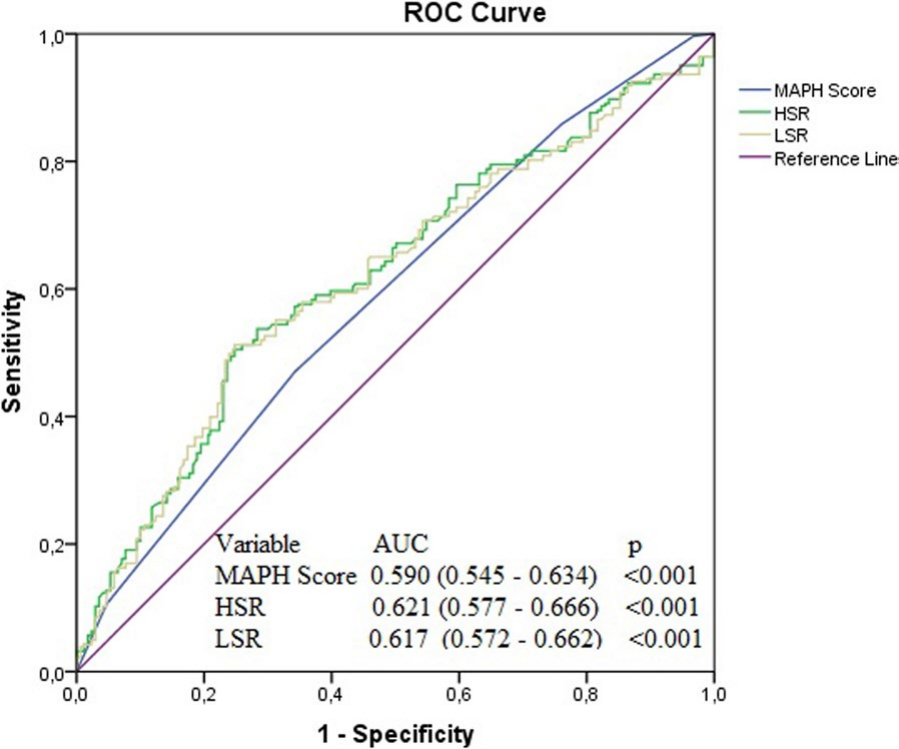
Receiver operating characteristics curve analysis of MAPH score, LSR and HSR in predicting high thrombus burden

The cut-off values for an HTB of age, MPV, serum total protein level and haematocrit for an LSR and an HSR were measured based on the Youden index (Fig. 1). The cut-off values determined were as follows: for MPV > 8.1 fL, sensitivity 57%, and specificity 55%; for age > 62 years sensitivity 55%, and specificity 56%; for haematocrit > 40.9%, sensitivity 64%, and specificity 53%; for serum total protein levels > 65 g/L, specificity 56%, and sensitivity 59%. In addition, the abilities of the LSR, HSR and MAPH score to forecast HTB were assessed by ROC curve analysis. The best cut-off value of the MAPH for forecasting HTB was 2 (sensitivity 67.9%, specificity 69.3%) based on the Youden index. The cut-off value HSR was 15.2 (sensitivity 72%, specificity 64%), and cut-off value an LSR was 40.3 (sensitivity 52%, specificity 75%) for predicting an HTB (Fig. 2).

## Discussion

Our main findings were that: patients with an HTB had a higher MAPH score than patients with an LTB, and the MAPH score was an independent predictor of an HTB. MAPH score at both LSR (0. 5 s^−1^) and HSR (208 s^−1^) was a strong and important predictor of HTB. To the best of our knowledge, this is the first study to research the confirmation of the MAPH score for predicting an HTB in patients with NSTEMI.

Numerous studies have been shown that intracoronary thrombus formation has primarily emerged due to plaque rupture (55–65%), followed by plaque erosion (30–35%) and calcified nodules (2–7%) [8]. Haemostatic plug formation that commonly arises at high blood shear rates and thrombosis due to endothelial dysfunction in vessels triggers fast platelet recruitment. Activation of the coagulation cascade begins quickly upon endothelial injury [8]. Blood viscosity is acknowledged to be an important component in thrombus formation [9]. Experimental studies have demonstrated that endothelial shear stress (ESS) affects the creation and progress of atheromatous plaque [10, 11]. The ESS is the shear rate multiplied by WBV. As the shear rate is a constant value, ESS can only increase with an increase in WBV [12, 13]. It has been reported that the higher WBV at both an LSR and an HSR is a significant determinant of thrombus burden in NSTEMI patients [14]. WBV is calculated with a previously validated formula using haematocrit and total protein levels [6, 15]. Abacıoğlu et al. reported that the MAPH score estimated the thrombus burden in patients with STEMI. In their paper, MPV levels and age were included in the calculation of MAPH score, in addition to total protein and haematocrit [4]. Abacıoğlu et al.’s [4] pioneering research found a relationship between blood viscosity and an HTB using the MAPH score in STEMI patients.

MPV is a measure of platelet size and is a key factor of platelet activity [16]. Increased MPV has been connected with poor clinical results in patients with various cardiovascular diseases [17, 18]. In our study, compared to patients with an LTB, MPV was significantly higher in HTB patients. We demonstrated that the MPV cut-off value of 8.1 fL may be a determinant of high thrombus load in patients with NSTEMI.

Age has been found to be an important determinant of morbidity and mortality in NSTEMI patients [19, 20]. Advanced age and an HTB were found to be associated with the risk of no reflow, in a meta-analysis of 27 retrospective and prospective studies [21]. In our study, age > 62 years was determined as the cut-off value for predicting an HTB. This finding is consistent with other reports that increased age was a risk factor for formation of thrombus and thromboembolic events [22].

A high thrombus load in NSTEMI patients has been reported to be associated with adverse clinical events such as the no-reflow phenomenon, stent thrombosis, and in-hospital mortality [14, 23]. In addition, the importance of blood viscosity has been revealed in previous studies. High WBV at an HSR and an LSR has been related to poor results for STEMI patients performed PCI with a risen risk for the presence of apical thrombus, stent thrombosis and no reflow [24, 25]. Moreover, elevated WBV at both shear rates has been related to an HTB and an independent predictor for an HTB in patients with NSTEMI [14]. Simone et al. [6] formulated simple equations using haematocrit and total protein levels for determination of WBV at various shear rates. The MAPH score, which calculated by adding MPV levels and age in addition to total protein and haematocrit, is a novel, easily accessible score.

The present study has a few limitations. The numbers of study population were low, and this study was planned as a single-centre and retrospectively. Thrombus grade was assessed based on only angiography images. Intravascular ultrasound or optical coherence tomography could not be utilised.

## Conclusions

The present study is the first study that investigates the association between the thrombus burden and MAPH score in patients with NSTEMI. MAPH score at both an LSR and an HSR was an independent predictor of high thrombus burden. Determining NSTEMI patients with a thrombus burden by utilizing this straightforward scoring tool may benefit to select the optimum management and decrease adverse outcomes.

## Data Availability

The datasets used and/or analysed during the current study are available from the corresponding author on reasonable request.

## Abbreviations

CAG: Coronary angiography
CI: Confidence interval
CHF: Congestive heart failure
DM: Diabetes mellitus
ESS: Endothelial shear stress
HSR: High shear rate
HTB: High thrombus burden
LSR: Low shear rate
LTB: Low thrombus burden
LVEF: Left ventricular ejection fraction
MPV: Mean platelet volume
NSTEMI: Non-ST segment elevation myocardial infarction
PCI: Percutaneous coronary intervention
STEMI: ST-segment elevation myocardial infarction
TIMI: Thrombolysis in myocardial infarction
WBV: Whole blood viscosity
